# Online Monitoring and Prediction of Thermo-Mechanics of AFP Based Thermoplastic Composites

**DOI:** 10.3390/s19061310

**Published:** 2019-03-15

**Authors:** Ebrahim Oromiehie, Nilanjan Das Chakladar, Ginu Rajan, B. Gangadhara Prusty

**Affiliations:** 1 ARC Training Centre for Automated Manufacture of Advanced Composites, School of Mechanical and Manufacturing Engineering, University of New South Wales, Sydney, NSW 2052, Australia; e.oromiehie@unsw.edu.au (E.O.); n.daschakladar@unsw.edu.au (N.D.C.); g.prusty@unsw.edu.au (B.G.P.); 2 School of Electrical, Computer and Telecommunications Engineering, University of Wollongong, Wollongong, NSW 2522, Australia

**Keywords:** fibre Bragg grating, automated fibre placement, laminated composites, thermoplastics

## Abstract

Precision sensing in the characterization of complex additive manufacturing processes such as the Automated Fibre Placement (AFP) technique is important since the process involves a significant level of uncertainty in terms of quality and integrity of the manufactured product. These uncertainties can be monitored by embedding optical fibre Bragg grating (FBGs) sensors which provide accurate and simultaneous measurement of strain and temperature during the AFP process. The embedded sensors have been shown to remain resilient in continuous health monitoring after manufacturing. The thermal history obtained from the FBG sensors demonstrates a reduction of temperature on the bottom ply by up to 25% when the plies are laid one above the other. A numerical tool is developed to identify the physical parameters which may be responsible for the rise/fall of the temperature during ply layup. The numerical findings agree well with the sensor data and is extended to capture a breadth of parametric studies through the layup simulation. The model provides a comprehensive insight to the characteristics of the laid and the laying ply from a thermo-mechanics perspective.

## 1. Introduction

The advent of smart, intelligent and adaptive materials and structures began in the mid-1980s with an attempt to integrate electroactive/optically active functional materials into large-scale structures in the form of in situ sensors and actuators. Composite materials which provide excellent light weight and stiffness characteristics in comparison to metals are often used to manufacture large structures. Such structures have gained impetus in the aerospace, renewable, marine and other manufacturing industries. The journey of composite structures began a while ago with manual hand-layup techniques which eventually slowed the rapid advancement in manufacturing. This is because of uncontrollable product integrity and a wealth of uncertainties. To improve the quality control, recent automation of the manufacturing process has increased the demand for composites and led to a significant rise in the global revenue. One such automation-enabled composite manufacturing is the automated fibre placement (AFP) process for making bespoke components [1]. The level of errors in the manual layup has been substantially overcome, yet the automated manufactured parts are still not free from defects. This requires efficient structural health monitoring (or in-situ product monitoring) during the AFP process to assess the quality of manufactured part. Monitoring of the quality in terms of their strain and temperature characteristics require precision sensors, suitable to capture information at high frequency and reliability. It means, the reliability must ensure the survival of the sensor in the measuring environment and consistency of data acquisition and performance.

The composite materials discussed in this article are primarily polymer composites reinforced with stiff fibres in a suitably engineered direction. The polymers can be of two types—thermosets (in which the polymers can cross-link together during thermal curing to form an irreversible chemical bond and sets itself due to the application of heat) and thermoplastics (where the polymer softens/melts and flows when heat is applied and exhibits a viscoplastic behavior under the thermal loading). The conventional method of monitoring the behavior of the composites is by strain gauges and a corresponding conditioning system [2,3]. In certain cases, the strain gauges are preferably melt-embedded within a thermoplastic composite to identify the formation of residual stresses (as a consequence of thermal and crystallization effects) [4]. At the onset of curing, custom-built thermal resistance strain gauges are used so that the residual stress-induced deformation can be measured. One such application is exemplified in case of the graphite/polyimide composites [5]. In general, due to the high processing temperature of thermoplastic composites (>300 °C), it is difficult to handle the strain gauges and trust the reliability of their performance [6]. As such if the characteristic and geometry of the embedding strain gauge does not merge with the laminate, it leads to a poor product quality and inappropriate measurements [6,7].

Limited literature is available for in-situ simultaneous measurement of strain and temperature during the AFP lay-up process. The ongoing issues with the use of strain gauges are their dimensions, and placement which require careful alignment of the sensor to avoid thickness variation of the final consolidated geometry. That is, a strain gauge or a thermocouple of certain dimensions is preferable if their geometry is within a close order of magnitude of the thickness of the composite tapes (~0.15 mm). Secondly, the measurements of temperature through strain gauge transducers are not accurate enough to rely on the data obtained during the manufacturing process. In this field optical fibre Bragg grating sensors (FBGs) stand out for in-situ structural health monitoring due to their localized and multiplexed sensing-capability [8]. First, the sensors are small in size (up to 125 μm), lightweight [9] and can be comfortably embedded within the laminates without affecting their integrity for continuous health monitoring [10]. In addition, they perform as a real-time multi-module data capture device for stress, strain, temperature, natural frequencies [11] and cracks [12]. In this article, we focus on the monitoring and prediction of thermoplastic composites during their automated layup process using FBG sensors.

The authors have successfully embedded FBG sensors for real time process monitoring and defect identification. Their initial study [13,14] characterized embedding of an array of two FBGs between the plies of a composite laminate. Due to the poor durability and highly brittle nature of the sensors, they were not brought into direct contact with the consolidation roller. That is, the measurement began from second ply upward to capture the effects of stacking plies, recovery time and residual strain/temperature. Wavelength was characterised to measure the performance of the FBGs, then calibrated and scaled to provide the necessary outputs. Another experimental program [15,16] considered an identification of misalignments through the embedment of these sensors within the composite laminate. It was concluded that the defect characteristic evolved different wavelength patterns in terms of wavelength profiles during heating and cooling, and wavelength shifts. In these studies, a combined effect of both consolidation pressure and curing temperature were measured for each lay-up and recorded as a wavelength change. Yet, the cross-sensitivity of FBGs to the strain and temperature was an issue which was addressed in the authors’ latest experimental study [17]. In this work, a new sensing head based on an angled FBG related to the straight one was utilized. The crux of this method is that it freezes the thermal sensitivity of angled FBG same as the straight FBG but flexes its strain sensitivity according to the orientation. Thus, using one set of embedded FBG sensor, both in-situ manufacturing process parameters and ex-situ structural health monitoring parameters can be measured.

The main objective of this study is to project to the scientific community how an accurately engineered coupled temperature-displacement analysis succeeds in predicting the temperature history measured with the help of an FBG sensor during a thermoplastic composite layup. This article is the first of its kind to simulate the FBG measurements in a strictly temperature-dependent manufacturing process and open a series of extensions in the field of prediction of smart monitoring systems. The article outlines two aspects of the entire objective—structural health monitoring (Section 2 and Section 3) and numerical assessment (Section 4) which is validated by the experimental findings.

## 2. Implemented Methods

### 2.1. Optical FBG Sensors

**Figure 1:**
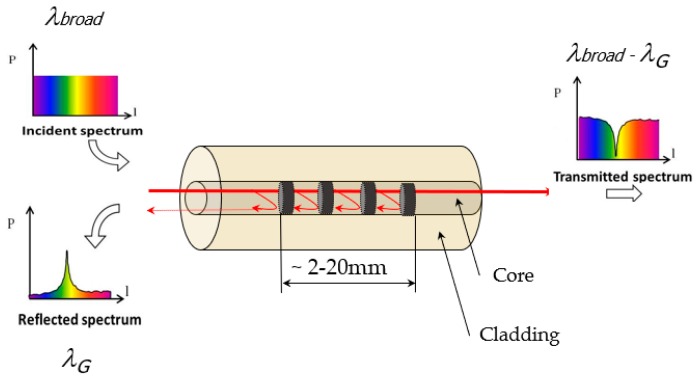
Working principle of an FBG sensor.

Optical FBGs are typically manufactured by exposing laterally the core of a single-mode optical fibre to a periodic pattern of intense UV light. The exposure causes a permanent change in the refractive index of the fibre’s core, creating a fixed and uniform index modulation (called as ‘grating’) relative to the exposure pattern. At each change of periodic refraction, optical signals are reflected which then combine coherently to one large reflection at a wavelength (called as the ‘Bragg wavelength’). This occurs at Bragg condition (Equation (1)) when the grating period is approximately half the input light wavelength:

(1) $$\lambda_{G}=2n_{eff}\Lambda$$

where *n_eff_* is the effective refractive index of the core and Λ is the periodicity of the refractive index modulation. The periodic index modulation enables the light to be coupled from the forward propagating core mode into backward propagating core mode generating a reflection response having a central Bragg wavelength *λ_G_* (Figure 1) [18]. The remaining light wavelengths which are not phase-matched are essentially transparent and come out of the core without reflection in the transmitted spectrum. Hence the ability to accurately adjust and maintain the grating wavelength is a rudimentary advantage of an FBG and the capability to inscribe multiple FBGs in an array format in a single optical fibre widens its scope. The reflected wavelengths, being a function of the refractive index and the grating period, which in turn is a function of the strain and the temperature becomes a suitable metric of monitoring strain-temperature response during the AFP layup process.

The basic principle of operation of an FBG-based sensor system is to monitor the strain and temperature-induced Bragg wavelength shift. The strain sensitivity of the Bragg wavelength arises from the change in period of the fibre coupled with a change in refractive index arising from the strain-optic effect, while the sensitivity temperature arises from the change in period associated with the thermal expansion of the fibre, coupled with a change in the refractive index arising from the thermo-optic effect. For the measurement of a temperature change, ΔT the corresponding wavelength shift is given by Equation (2):

(2) $$\Delta \lambda_{T}=\lambda_{G}\left(\alpha +\xi \right)\Delta T,$$

where α is the coefficient of thermal linear expansion of the fibre material and ξ is the fibre thermo-optic coefficient.

The wavelength shift, Δ*λ_S_*, for the measurement of an applied uniform longitudinal strain, Δ*ε*, is given as Equation (3):

(3) $$\Delta \lambda_{s}=\lambda_{G}\left(1-\rho_{e}\right)\Delta \varepsilon ,$$

where $\rho_{e}$ is the photoelastic coefficient. For a silica core fibre the value of $\left(1-\rho_{e}\right)$ is usually 0.78. Thus, the measure of the wavelength shifts determines the changes in temperature or strain. The typical temperature and strain sensitivity of a silica FBG in free space is ~10 pm/°C and 1.2 pm/µε, respectively. When embedded inside a composite, due to the thermal expansion of the material, the strain and temperature sensitivity of the FBG sensor will differ from this value. Details of the FBG sensors and their configuration are discussed in the methodology part of Section 3.

**Figure 2:**
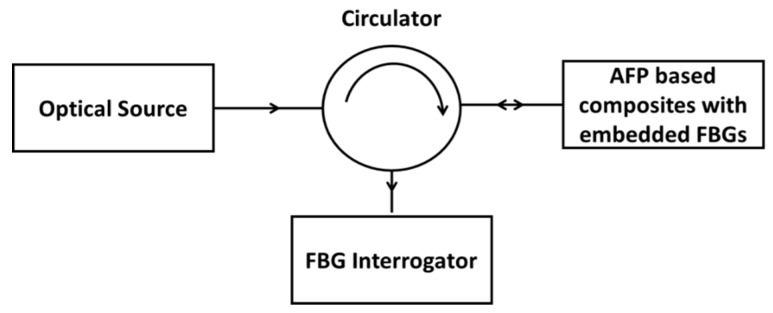
Schematic of an FBG sensor-based characterization system.

The FBG interrogation system used in this study is IMON 256 from Ibsen Photonics A/S (Farum, Denmark), which has a capability to resolve 5 pm wavelength change with a data acquisition rate of 3 kHz is used to acquire the data from the FBG sensors. A broadband source with a spectral range of 1530–1570 nm was used and the reflected signal from the FBG was directed to the interrogator via fibre optic circulator, shown by a schematic (Figure 2).

### 2.2. Automated Fibre Placement (AFP) Methodology

**Figure 3:**
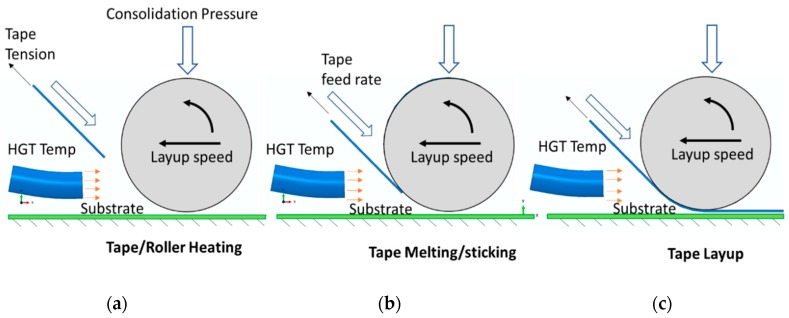
Three steps of the layup process. (**a**) HGT heats the bottom of the roller; (**b**) Tape/Strip accelerates to the bottom of the roller while being heated by HGT; (**c**) Roller consolidates the tape and lays on the substrate.

The AFP layup is an automated manufacturing process of making composite structures which employs several individual subsystems to coherently work together in the process and are carefully installed in the placement head. The machine has three main components—a robotic arm, a placement head and a computer controller. The robotic arm allows translation and rotation in three mutually perpendicular directions, relative to a linear or rotary travel of the substrate. The important component of all is the placement head which is carefully engineered for thermoset or thermoplastic materials. The head comprises of a consolidation system (that is, a polymer roller for thermosets and a stainless steel roller for thermoplastics, the choice of roller material depends on the process temperature of the composite), tape feeding and chopping system (up to four rolls of tapes are held at the top which are fed through a guide and are chopped off, when needed, through a program or manual override) and the heat source (a relatively low temperature in case of thermoset to have enough tackiness to bond and a high temperature hot gas torch (HGT) for thermoplastics to melt and weld) (Figure 3). The AFP robot lays up a composite tape simultaneously heating and consolidating on a substrate or on a previously laid tape. This means, a wealth of AFP parameters influences the quality and integrity of a laminated composite which include the HGT temperature, the hot gas flow rate, the consolidation pressure, the steering of the robot head/tow and the lay-up speed/deposition rate. As mentioned earlier, the temperature is a prime factor for thermoplastic consolidation and melting, and this article mainly focuses on thermoplastic AFP layup and its monitoring and assessment of strain-temperature through FBG sensors.

## 3. Experiments

### 3.1. Specimen Preparation

Specimens were manufactured employing AFP which contain ten plies (one above the other) of unidirectional CF/PEEK (AS4/APC2) prepreg. The material properties of the prepreg tape are provided in Table A1, Table A2 and Table A3. The prepreg had a fibre volume fraction of 60% with a thickness of 0.15 mm and width of 12.7 mm. The process temperature of this thermoplastic reaches up to 350 °C (where 345 °C is the melting point of the polymer of the prepreg which ensures that the tape completely melts before laying up). The polyimide coating on the FBG sensors withstands the same temperature and is reliable in measuring the temperature history during the process.

**Figure 4:**
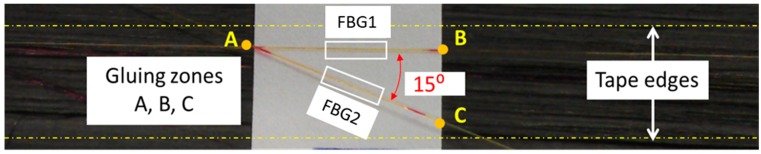
The aligned FBGs above the 2nd ply.

In this experiment, the standard apodized polyimide coated FBGs of cladding diameter of 0.125 mm and grating length of 10 mm were used. The FBGs had a peak reflected wavelength of 1549.6 nm and 1554.5 nm and a reflectivity greater than 90%. When the sensors were attached on the 1st ply they were damaged during the layup of the 2nd ply while in contact with the hot compaction roller. Subsequently the sensors were attached on the 2nd ply and experimental measurements began during the layup of 3rd ply to 10th ply upward, however, the measurements for the 3rd ply were discarded due to noise and disturbances. It is believed, that during the layup of the 3rd ply the compaction pressure by the roller on the sensors (which were attached on the 2nd ply) was enough to destabilize the alignment of the sensors which were not noticed in the subsequent layups. As a result, the experimental results were considered from the 4th ply upward [17]. The sensors were glued on the 2nd ply with the help of commonly available, Methyl 2-Cyanoacrylate (indicated as gluing zones in the Figure 4).

### 3.2. Strain and Temperature Measurement

In this scheme, the angled and straight FBGs will have distinct strain sensitivities due to their orientation with the composite laminates, while retaining the same temperature sensitivity. The angle between the FBG sensors was set to 15° (refer to Section 3.1). The strain (Δε) and temperature (Δ*T*) at the vicinity of the FBG sensors can be calculated using the coupled characterization matrix [17] as shown in Equations (4) and (5):

(4) $$\left[\begin{array}{c} \Delta T\\ \Delta \varepsilon \end{array}\right]=\frac{1}{D}\left[\begin{array}{cc} \kappa_{\varepsilon 2} & -\kappa_{\varepsilon 1}\\ -\kappa_{T2} & \kappa_{T1} \end{array}\right]\left[\begin{array}{c} \Delta \lambda_{FBG1}\\ \Delta \lambda_{FBG2} \end{array}\right],$$

(5) $$D=\kappa_{T1}\kappa_{\varepsilon 2}-\kappa_{\varepsilon 1}\kappa_{T2}$$

where Δ*T*, Δε and Δ***λ*** are in degree centigrade (°C), micro-strain (με) and nanometres (nm) respectively. κ_ε_ and κ_T_ are the strain and thermal sensitivities of FBGs. As mentioned in Section 2.1, the strain and thermal sensitivity of the free space FBGs in the normal conditions are 1.2 pm/με and 10 pm/°C respectively. Once embedded inside the composites, the strain sensitivities are expected to be different from that of the free space one. This requires measurement of the true strain sensitivity of the FBG sensors.

**Figure 5:**
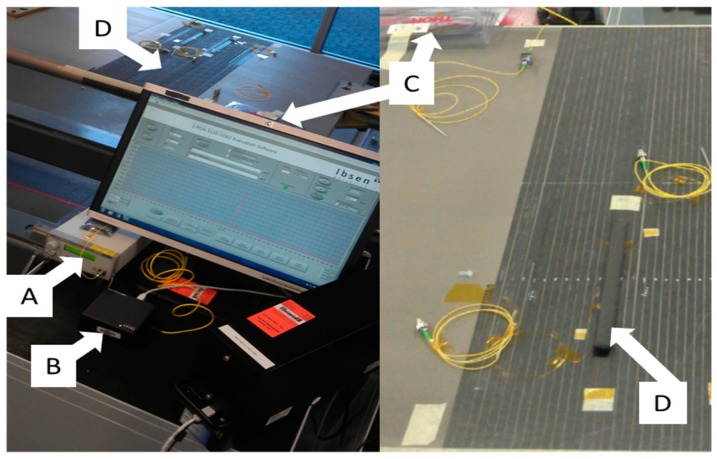
Experimental measurement of strain and temperature during the lay-up process in AFP (A: Broadband Source, B: Interrogator, C: Circulator, D: Laminate with embedded FBGs).

The strain sensitivity of the straight and angled FBGs were obtained experimentally as 1.16 pm/με (*κ*_ε1_) and 1.9 pm/με (*κ*_ε2_), respectively. Figure 5 illustrates the experimental set up during the AFP lay-up process using photonic technologies, where, A, B, C and D are the Broadband Source, the Interrogator, the Circulator, the Laminate with embedded FBGs. The polyimide fibres containing the FBGs are fusion spliced to a fibre optic pigtail (yellow coloured fibre cable) which connects to the interrogator. The spectral variation of the reflected signal from the FBGs was monitored during the entire fabrication process for each ply from second ply upward. The HGT was set to a temperature of 750 °C to reach the process nip point temperature of 350 °C to melt the PEEK composite tape.

With the measurement of the wavelength changes for the straigth (Δ*λ*_FBG1_) and angled (Δ*λ*_FBG2_) FBGs, for each lay-up, and substituting them in the Equations (4) and (5) the exact values for the strain (Δε) and temperature (Δ*T*) were presented in [17].

## 4. Numerical Simulation

Modelling of AFP of thermoplastic composites involves simulation of physical processes employing non-linear thermal and mechanical interactions which allow the thermoplastic to melt/soften in the process and act as a medium to lay and bond high stiff carbon fibres under controlled consolidation. A wealth of literature is available on estimating the degree of bond and crystallinity during thermoplastic consolidation which considered the mathematics of viscous flow of the polyether ether ketone (PEEK). These studies simplified the calculations of the bonding by assuming the roughness of the bottom tape in the form of square wave such that the square peak of the laid tape meets the same of the top laying tape in the process [19,20,21,22,23,24]. The bonding continues until the assumed squared geometry of the peak flows, due to application of heat and pressure, laterally to an extent until autohesion is established [25,26,27,28,29,30,31,32,33,34]. In addition to the viscous characteristics of the thermoplastic, the tape may exhibit non-linear elasticity in the compaction direction from a multiscale perspective [35] as a function of inter-fibre friction effects [36]. To predict the performance of such a composite part and its as-manufactured quality, process model is important where repeated experiments are prohibitive. Process modelling offers a scientific understanding between process variables and the material behavior with the help of established theory applied to complex manufacturing environment, such as AFP. It also allows an efficient optimization of process variables to achieve a desired characteristic of the part. Some studies have considered sequential heat-transfer and structural analysis to simulate the AFP layup process but loses the inter-relationships in the science of thermo-mechanics of the process and hence cannot be fully relied [37,38,39,40,41,42,43]. The current study establishes a simplified coupled temperature-displacement analysis of layup of four thermoplastic carbon plies laminate being consolidated by a stainless steel roller. The model assumes a linear elastic and temperature-dependent orthotropy of the thermoplastic material. The aim of the model is to compare the thermal history as obtained from the FBG Sensors and exercise further capabilities and robustness of the model.

### 4.1. Coupled Temperature-Displacement Analysis

Problems involving effects of heat transfer on the structural dynamics of a system and vice-versa are modelled either using a sequentially coupled thermal stress analysis or a coupled temperature-displacement analysis. The sequentially coupled analysis is efficient, yet it lacks the ability to accurately interpret the interdependence of the thermal and the structural analysis and hence, a fully coupled thermo-structural analysis stands out and is used in the current study.

In case of transient dynamics, the governing equation of a multi-degree of freedom system can be cast in a nodal matrix form as in Equation (6):

(6) $$\left[M\right]\left\{\ddot{u}\left(t\right)\right\}+\left[C\right]\left\{\dot{u}\left(t\right)\right\}+\left[K\right]\left\{u\left(t\right)\right\}=\left\{f\left(t\right)\right\}$$

where ***M***, ***C***, and ***K*** are the mass, damping and stiffness matrices of the dynamic system, family of *u*(*t*) represents the displacement, velocity and acceleration of the nodes of the system with respect to time, *t*, and ***f***(*t*) are the nodal equivalent forces in a cartesian coordinate system. The transient solution is obtained by using the time marching scheme derived by the Newmark integration for displacement and velocity, coupled with the damping matrix, ***C*** identified through the Hilber, Hughes and Taylor’s implicit operator [44,45].

In case of a transient heat transfer analysis of a homogenous anisotropic material, the generalized heat diffusion equation is Equation (7):

(7) $$\rho c_{p}\frac{\delta T}{\delta t}=\nabla \left(k.\nabla T\right)+f_{source}$$

where ρ is the density, $c_{p}$ is the specific heat capacity at constant pressure, ***k*** is the vector of thermal conductivities, *T* is the temperature. As the layup strategy demonstrates the placement of the tapes in the through-thickness direction (z-axis), a thermal boundary condition in this direction while laying up a tape over a bottom tape can be expressed as in Equation (8):

(8) $$q_{z}\left(t\right)=k_{z}\frac{\delta T}{\delta z}=-h_{conv}\left(T_{2}-T_{room}\right)+f_{source}\left(t\right)+h_{contact}\left(T_{2}-T_{1}\right)$$

where, $k_{z}$ is the conductivity of the tape in the thickness direction, $\frac{\delta T}{\delta z}$ is the temperature gradient, $h_{conv}$ is the convective heat transfer coefficient (negative sign preceding the convection term denotes the heat is escaping out of the system), $T_{2}$ is the temperature of the tape being laid over the bottom tape which is at temperature $T_{1}$, $T_{room}$ is the room temperature, $f_{source}$ is the heat flux entering into the system through the HGT at the bottom of the tape and the consolidation roller at the region of contact, $h_{contact}$ is the thermal conductance at the contact between the top and bottom tape (the thermal contact conductance vanishes for the first tape placement and is activated from the second tape upward). The radiative heat loss to the surroundings is also ignored in Equation (8) for simplification of the present study.

While coupling these two procedures, one could observe that there will be an influence of the heat transfer analysis onto the mechanical stress, primarily from two aspects—(i) at a calculated nodal temperature, the correct magnitude of temperature-dependent stiffness of the tape is mandatory, and (ii) at a calculated nodal temperature gradient, thermal strains need to be recomputed with the correct magnitude of temperature-dependent coefficient of linear expansion of the thermoplastic material. This facilitates the current work to utilize the benefits of a coupled temperature-displacement analysis procedure. Two categories of implementation of such procedures are available—exact and approximate. The exact implementation involves a non-symmetric Jacobian represented by the coupled equations (Equation (9)), where Δu and ΔT are the incremental adjustments to the displacement and temperature, $K_{ij}$ are the factored stiffnesses of the Jacobian and $R_{u}$ and $R_{T}$ are the residual vectors for the mechanical and thermal analyses which are used to solve a finite element problem. The approximate implementation scheme evolves the mechanical and the thermal solutions simultaneously but with a weak interdependence between the two solutions. That is, the off-diagonal entries of (Equation (9)) are carefully neglected, implying a faster convergence of the solution. The approximate implementation scheme of the fully coupled thermal stress analysis has been used in this study:

(9) $$\left[\begin{array}{cc} K_{uu} & K_{uT}\\ K_{Tu} & K_{TT} \end{array}\right]\left\{\begin{array}{c} \Delta u\\ \Delta T \end{array}\right\}=\left\{\begin{array}{c} R_{u}\\ R_{T} \end{array}\right\},$$

### 4.2. Modelling Strategy

The numerical study characterized the layup of four AS4/APC2 PEEK carbon tapes of 12.7 mm width and 0.15 mm thickness. A length of 12 mm is used in the analysis, the selection of this length is explained later. The objective of the study is to interpret the physics behind the tape’s thermal deformation and mechanics of consolidation, with the help of a commercial finite element package, ABAQUS. Since the modelling of the thermoplastic flow, roller consolidation and tape placement would be computationally prohibitive, hence, a two-stage model is conceived—(i) identification of the contact stress contour and deformation during the consolidation and, (ii) application of the obtained contact stress contour to simulate a realistic tape consolidation. Temperature-dependent mechanical and thermal properties of the tape and the consolidating roller is provided in Table A1, Table A2, Table A3 and Table A4.

### 4.3. Finite Element Methodology

In the first phase, a thermomechanical contact study was carried out between the roller and the thermoplastic tape. The contact stress results were compared with the analytical Hertz solution [46] which assumed elastic, isotropic and frictionless contact between two cylindrical bodies (in this case, the stainless steel roller which has a finite diameter and the flat tape with an assumed infinite diameter). The Hertz solution can be approximated to a nominal line contact between two cylinders whose axes are parallel to each other. The surface stresses, half-contact width, and peak pressure can be expressed as Equations (10)–(12):

(10) $$p\left(x\right)=p_{0}{\left\{1-\frac{x^{2}}{b^{2}}\right\}}^{\frac{1}{2}}$$

(11) $$b=\sqrt{\frac{4F\left(\frac{1-\nu_{R}^{2}}{E_{R}}+\frac{1-\nu_{T}^{2}}{E_{T}}\right)}{\pi L\left(\frac{1}{R_{R}}+\frac{1}{R_{T}}\right)}}$$

(12) $$p_{0}=\frac{2F}{\pi bL}$$

where, the p(x) is the contact stress along the *x* direction (i.e., transverse to the load along *y*), *b* is the half-contact width (given by Equation (11)), *F* is the applied load (=180 N consolidation force by the roller on the tape), *L* is the width of the roller (assumed equal to the width of the tape), *E*, υ and *R* denote the elastic modulus, Poisson’s ratio and radius of the roller and the tape with respective subscripts as *R* and *T*, and $p_{0}$ is the peak pressure of the contact ellipse. The material properties of the roller and the tape are taken from Table A1, Table A2, Table A3 and Table A4 and for the Hertz analytical solution, an elastic modulus of 10 GPa has been considered for the tape material, which is in order of the magnitude of the transverse modulus of the tape.

**Figure 6:**
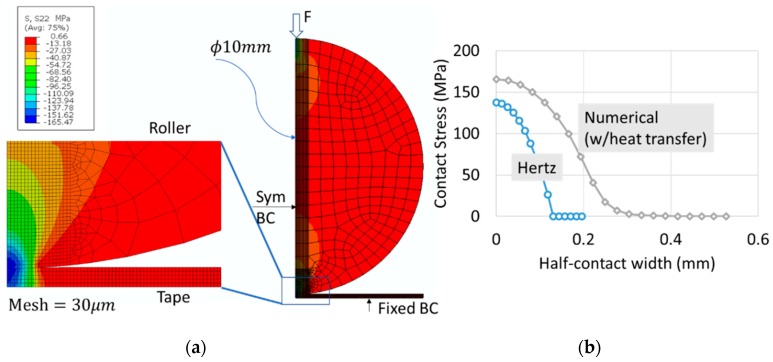
(**a**) Two-dimensional plane strain analysis of contact between roller and tape, (**b**) Comparison of contact stress profile with Hertz analytical solution.

A two-dimensional plane strain geometry of the roller and the tape was considered, which were meshed with 4-node plane strain quadrilateral element with temperature degree of freedom (Abaqus type, Constant Plane Strain 4 node with temperature degrees of freedom, CPE4T). An average element size of 0.5 mm with fine meshing of 0.03 mm at the contact region to simulate the elliptic contact contour. Figure 6 illustrates the contact stress contour, represented by the stress in the loading direction (in this case, Abaqus stress variable in y direction (in this case ‘S22’)) to identify the peak pressure (in this case, 165 MPa) and the half-contact width (~0.6 mm) in presence of thermal diffusion and expansion. The left edge of the roller and the tape are applied symmetric boundaries with the bottom of the tape fixed (assuming it does not slip relative to the substrate). A magnitude of 0.2 W/mm^2^ of heat flux is applied to the bottom of the tape which matches the nip point temperature.

Equations (10)–(12) are used to find out the equivalent Hertz contact stress and compared with the numerical solution (Figure 6). The simplified model of Hertz shows a reduced peak pressure of 137 MPa in comparison to 165 MPa, because of the thermo-mechanical deformations of the tape and the roller and the material orthotropy of the tape. This has increased the Hertz half-contact width from 0.2 mm to up to 0.6 mm, signifying the influence of heat transfer to Hertz solution. The results imply that to simulate the layup of a thermoplastic tape the mechanics of consolidation cannot be simplified using a conventional Hertz solution and it is recommended to identify the peak pressure and contact width based on a thermomechanical analysis.

In the second phase, the contact stress information obtained from the first phase of numerical study is applied as a pressure load during the layup. This simplifies the consolidation process by omitting the need of modelling the geometry of the roller. The roller was replaced by a localized contact stress profile (Figure 6) over a calculated contact width (in this case, 0.6 mm × 2 = 1.2 mm). The time taken by the roller to traverse this width is based on its deposition or layup speed (in this case, 76 mm/s) which is equivalent to 16 ms. In terms of applying heat flux, it is carefully noted that the bottom of the tape is directly heated by the HGT to a magnitude of heat flux close or equal to the melting point of the PEEK such that the material melts/softens to initiate bonding with the substrate or the bottom layer. Once the roller consolidates the tape and leaves a contact width, the top surface of the tape (which was under the roller) immediately begins to convect and radiate the heat, cooling the temperature of the laid tape. It can well be conceived that the convection and/radiation from the bottom and rest of the edges of the laid tape is insignificant relative to the top surface of the tape. The convective heat transfer coefficient, in case of PEEK, was expressed in terms of characteristic element length [29] and can be calculated using Equation (13), where *h*, Δ*T, L* are the magnitude of the convective heat transfer coefficient (W/mm^2^ K), temperature difference (K), and element length (mm):

(13) $$h=1.32{\left(\frac{\Delta T}{L}\right)}^{\frac{1}{4}}$$

**Figure 7:**
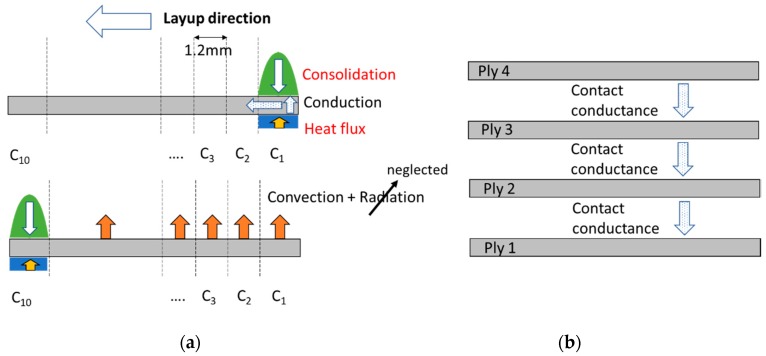
(**a**) Pressure and heat flux boundary during ply layup, (**b**) Contact conductance between the laid and laying plies.

In terms of boundary conditions, the bottom of the first tape is restricted to move in the upward direction, which simulates the ply fully bonded with the metal substrate. A half-width (=6.35 mm) geometry of the tape is created with the left edge as a symmetric boundary and the edge of the tape at the start of the layup assumes a perfect bond with the substrate. An Abaqus type, C3D8T (full integration 8-node linear brick element with temperature controls) and an element size of 0.3 mm is used to mesh the ply with a further element seed of 0.1 mm across the thickness of the tape, to capture the through-thickness behaviour. Figure 7 illustrates the layup simulation of each ply from left to right which is divided into ten contact widths, C*_i_* (*i* = 1, 2,…, 10) and the consolidation and heat flux is applied to each of C*_i_* in course of roller movement. On the right of Figure 7, shows the thermal contact conductance between the plies as the heated bottom of the laying ply meets the cooled top (which was under convection) of the laid ply.

In terms of simulation, the sensor temperature is assumed to be the temperature at the top surface nodes of the 1st ply. Due to the identical geometry and material properties of each ply in the simulation, a numerical analysis of four plies from 1st to 4th ply will be a representative of the experimental results from 4th to 7th ply (discussed in Section 3.1). Based on the accumulated effects of geometry, material and thermal nonlinearities on the total simulation time to lay up a single ply (detailed in Section 5), up to four plies were chosen to represent the trend of the experimental findings and understand the underlying thermo-mechanical concepts. This remains valid, until a stochastic variability in ply material properties and a CT-generated geometry of each ply are considered to represent the true picture of ten plies.

A magnitude of length equal to ten contact widths that is, 12 mm is considered for each ply including a half-width of 6.35 mm and thickness of 0.15 mm for the simulation. All the plies are initially modelled in contact with each other, but the contact algorithm is activated once a ply layup begins which indicates a realistic layup process. Uniform meshing as discussed above is carried out on all the plies. The contact between two plies are modelled with care. The laying ply is assumed as the master surface over the bottom ply (the slave). This is because, the small thickness of the laying ply (=0.15 mm) will allow the heat to conduct and diffuse into the laid ply which is also observed in the experimentally observed temperature characteristics of the laid ply. The numerical heat transfer allows generation and dissipation of heat during contact through the thermal conductance. In terms of dimensionality, the unit of thermal contact conductance (W/mm^2^ K) is different to conductivity (W/mmK) since the latter is conceived through a material and the former through the material contact interfaces. It is to be noted that when the third ply is laid, the contact conductance not only occurs between second and third but between first and second as well if there is an appreciable temperature gradient at their contact interface. Sensitivity studies on the contact conductance and the heat flux can accurately dictate the temperature characteristics of all the plies. Since the plies come in perfect bond during consolidation, the frictional slip is ignored, and the bottom ply nodes are not allowed to slide once in contact with the top ply nodes. The prediction of the degree of bond and change of crystallinity has been kept out of the scope of the current study.

## 5. Results and Discussion

All the models were run on a 16 GB Ram, quad core processor and took a simulation time of 40 min per ply layup for a four-ply stack. This section demonstrates the effects of mesh size, heat flux, thermal contact conductance and comparison of thermal history from the numerical results with the experimental data.

### 5.1. Mesh Sensitivity

**Figure 8:**
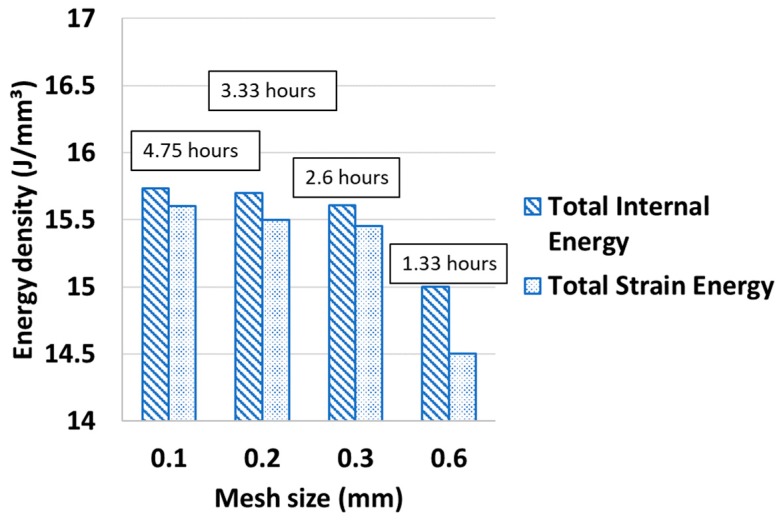
Effects of mesh size on strain and internal energy.

Mesh resolution is an important parameter for a numerical analysis in order to assess the effect of degrees on the response of the system. In case of a coupled temperature-displacement analysis, there are two metrics to decide the sensitivity of mesh—total strain energy (due to mechanical stresses) and total internal energy (due to thermal stresses) (Figure 8). With the reduction of the mesh size (or increase in degrees of freedom) the strain and internal energies, expressed in terms of their energy density, becomes gradually steady but at the cost of computational time. The time taken to simulate across the mesh sizes are shown in boxes above the column chart. An increase from 15 J/mm^3^ to 15.7 J/mm^3^ of strain energy and 15.4 J/mm^3^ to 15.8 J/mm^3^ of internal energy are observed for a decrease of mesh size by six times. Based on the mesh sensitivity study, the rest of the simulations are carried out with a selected mesh size of 0.3 mm.

### 5.2. Effects of Heat Flux

**Figure 9:**
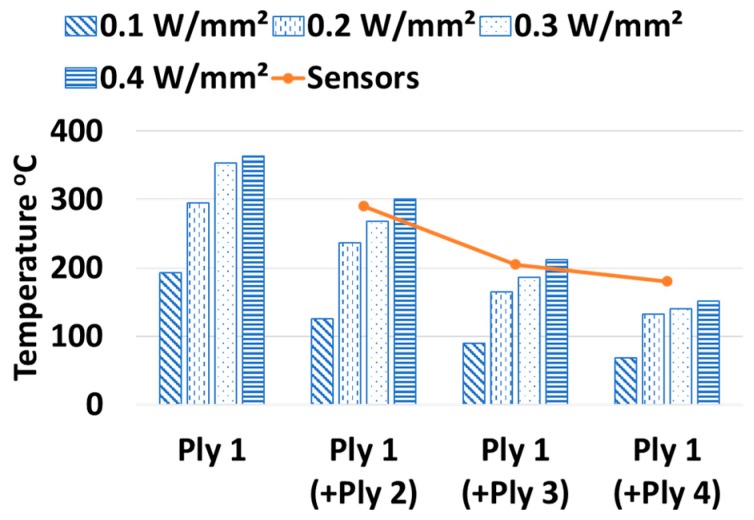
Effects of heat flux on laid ply.

**Figure 10:**
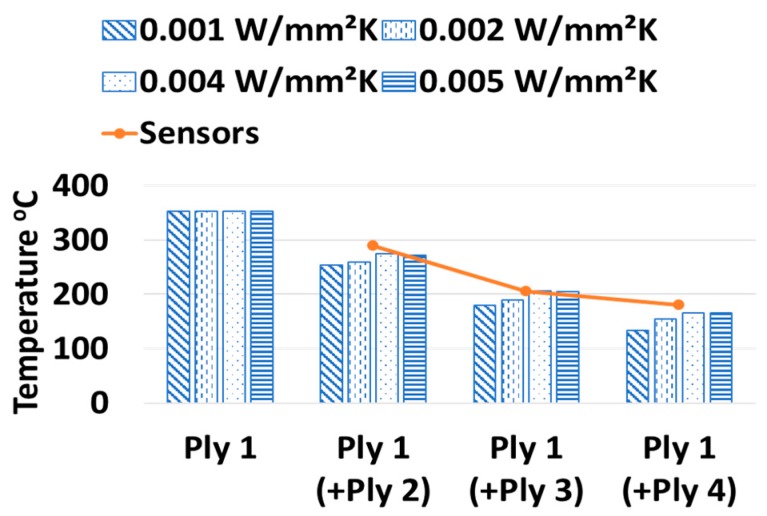
Effects of inter-ply thermal contact conductance on laid ply.

In case of numerical prediction of a thermal system, the parameter that directly decides the rise or fall of temperature at a targeted node is the heat flux. In the layup of AFP-based composites, the HGT rises the temperature at the bottom of the tape to its melting point. A magnitude of heat flux is correctly chosen to represent the temperature of the plies, obtained by the sensors during the ply layup. Figure 9 demonstrates the effects of different magnitude of heat flux on the temperature of the bottom ply as new plies are laid over it. It is seen that a linear increase of heat flux causes a proportional rise in the temperature. This is because, the heat flux is applied uniformly at the bottom of the laying ply. In the figure, the temperature of the bottom ply is plotted across the layup number, that is, in the abscissa, Ply 1(+Ply 3) means the temperature of the Ply 1 as the Ply 3 is laid on the top. The agreement of the numerical findings with the sensor-acquired data compares well for Ply 1 and lies between the has been chosen words missing as 0.2 W/mm^2^ for rest of the study. The slight deviation between the simulated and the experimentally obtained temperature is attributed to the idealized symmetric boundaries and uniform thermal load at the bottom of the laying ply. This is because, the temperature degree of freedom at the boundary nodes directly depends on the type of boundaries used and the effect of heat flux on the nodal temperature is not linear since the heat is at the same time convected through a heat transfer coefficient (Equation (13)) to ambient and conducted through a thermal contact conductance (Figure 10) to the bottom ply. So the precise magnitude of the heat flux would lie somewhere between 0.2 and 0.3 W/mm^2^ and would vary nonlinearly if the number of plies increase. An accurate representation of the nodal boundary would require a nonlinear contact stiffness between the laid and the laying ply and a time-temperature dependent nodal stiffness at the boundary in place of a fixed boundary condition.

### 5.3. Effects of Thermal Contact Conductance

The high temperature used to melt the bottom of the laying tape, when it meets the top of the laid tape, dissipates/conducts heat across the interface between the laying and the laid tapes. This heat conduction across the inter-ply interface is simulated with the help of thermal contact conductance which acts on per unit area of the interface. Figure 10 illustrates the temperature of the Ply 1 because of other plies. There was no ply over Ply 1 during its layup, hence due to no contact, the temperature estimated on Ply 1 had no effect on the thermal conductance. In the rest of the cases, a value of thermal conductance between 0.002 and 0.004 W/mm^2^ K demonstrated well in simulating the ply temperature because a specific value of the thermal contact conductance cannot be ensured due to nonlinearity of heat dissipation during conduction and convection.

### 5.4. Numerical vs Experimental Thermal History

**Figure 11:**
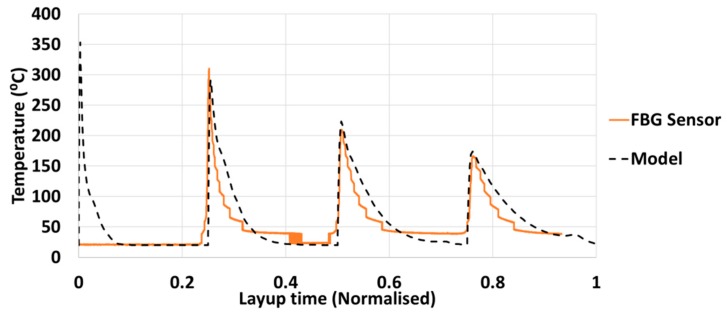
Comparison between numerical and experimental results.

The thermal history of the bottom ply is compared with the sensor acquired data, discussed in Section 3 (Figure 11). The bold line represents the FBG sensor data, followed by the predicted results in terms of a dashed line. The effective ply length in case of test was 304 mm and in case of simulation was 12 mm (the length obtained from analytical thermomechanical calculations). In both the cases, the layup speed was 76 mm/s so the lay time in test was 4 s and in simulation was 0.16 s. That is why, a suitable normalization of the layup time was necessary to compare the gradient of AFP heating and cooling cycle. In this case, it was achieved by dividing the simulation and the experiment time by their respective total layup times which brings the corresponding time scale between 0 and 1. Thus, the layup time for the experiments and the numerical simulation are normalized by dividing the total layup time, because of the difference in length of the plies in the test and the simulation. The peak temperature of the bottom ply in both the cases is found to reduce as new plies are laid, this is because away from the bottom ply more heat is dissipated and conducted to the adjacent neighbors, leaving limited flow to the bottom. No data is obtained from the sensor at the initial ply, since the sensor broke during consolidation and has been avoided from measurement until the next ply is laid. However, in case of the model, there was no such discrepancy and the first data are registered. As expected, the chosen heat flux (=0.2 W/mm^2^) melts the bottom of the ply (raises the temperature above the melting point of PEEK, 345 °C) and immediately begins to convect as the heat source is removed. On placement of above plies, this temperature continues to reduce, until solidification (or crystallization) begins from the bottom plies. Another observation was made from the difference in the test and the model, is that upon convection, the ply temperature during the test does not reduce fully to room temperature (20 °C) but remains at a little higher to a value of 40 °C. This may be attributed to uneven cooling of the ply and its material and geometric non-linearities.

### 5.5. Overall Thermal History

**Figure 12:**
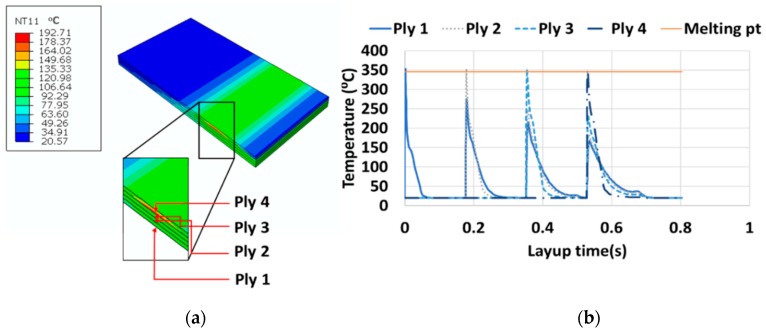
(**a**) Contour of temperature across the four plies, (**b**) Thermal history of all the four plies.

The thermal history of all the four plies are plotted in Figure 12. The contour plot on the left illustrates the temperature distribution (in this case, Abaqus variable, NT11—Nodal temperature; the unit of temperature is added in °C in the Abaqus generated legend since the software does not produce legend unit and works based on a set of consistent units) during the 4th ply layup and its effect on the temperature of the remaining plies. The limit of the contour demonstrates the lower range of the temperature of all the plies is about 21 °C (shown in blue) which is higher than the room temperature and the upper range at a layup time is 193 °C at the bottom of the 4th ply shown in red. The plot shows that all the plies are heated above the melting point and subsequently the heat dissipates reducing the temperature to room temperature.

## 6. Conclusions

A combination of straight and angled FBG sensors was implemented to simultaneously monitor the strain [17] and temperature during an automated fibre placement of thermoplastic carbon tape. In the experimental set up, ten plies were laid one above the other and up to four plies were simulated to investigate the thermo-mechanics during the layup process. Care was taken to measure the strain and temperature sensitivities which were used to determine the absolute variables during the AFP process. Next, the underlying physics of the thermoplastic melting and consolidation was captured through a numerically coupled temperature-displacement model which compares with the experimental findings with a reasonable agreement.

As the consolidation roller passes over the FBG sensor, the sensors are axially strained both due to the actions of mechanical and thermal strain (because of the heating by the HGT). This causes a permanent change in the reflected wavelength which is proportionally scaled to obtain the strain and temperature information. The response indicates a loading-unloading cycle during each ply layup. The gradient of the load phase is steep since the roller or the laying ply is in contact with the sensor on the bottom ply for a small period. This is followed by a gradual convection or release of the stresses, which is reflected by a smoother unloading of the strain-temperature history. Secondly, more the number of plies laid on top of the sensing ply, the peak temperature gradually reduces, implying more heat is conducted to the plies above and rest dissipated to the surroundings. The major challenges faced during data acquisition from these FBG Sensors are—(i) the sensor survival, and (ii) the incompatible adhesion between the sensor and the laid tape.

A process model is developed with an aim to understand the thermo-mechanics of the AFP process. Previous studies have simplified the predictions by using a sequential heat transfer approach, and therefore, lacked the interdependence of the coupled governing equations. The current model considers a fully coupled thermo-mechanical analysis to identify and predict the response history of the AFP process variables. Mesh sensitivity studies demonstrated that a mesh size of 0.3 mm portrays the true picture of the AFP process at a reasonable expense of simulation time. The magnitude of heat flux has a significant effect on the ply temperature. Heat flow at the contact between the laying and the laid tape is numerically simulated as an effect of the thermal contact conductance. A specified value of which cannot be ensured due to nonlinear dissipation of heat during conduction and convection, a value between 0.002 W/mm^2^ K and 0.004 W/mm^2^ K concludes closer approximation of the temperature obtained by the sensors. A comparison of the numerically estimated temperature history matches well with the history captured by the FBG sensor. The model presents a baseline predictor of the AFP layup simulation which gives a direction to further investigate the microdetails of the process.

## Appendix A

**Table A1:** Temperature-dependent density and specific heat of APC2 PEEK.

Temperature T (°C)	Density *ρ* (kg/m^3^)	Specific Heat C*_p_* (J/kgK)
0	1601	800
50	1598	930
100	1593	1040
150	1586	1260
200	1575	1300
250	1563	1400
300	1551	1550
350	1537	1650
400	1524	1700

**Table A2:** Temperature-dependent elastic properties of APC2 PEEK.

Temperature T (°C)	Elastic Modulus E_11_ (GPa)	Elastic Modulus E_22_ = E_33_ (GPa)	Poisson’s Ratio υ_12_ = υ_13_ = υ_23_	Shear Modulus G_12_ = G_13_ (GPa)	Shear Modulus G_23_ (GPa)
23	130	10.3	0.32	6.00	4.8
65	130	9.6	0.33	5.43	4.34
121	130	8.3	0.32	4.86	3.89
168	127	4.3	0.34	2.51	2.01
182	126	4.3	0.34	2.16	1.73
232	125	3.6	0.4	0.951	0.761
288	125	1.7	0.4	0.531	0.425
315	124	0.63	0.4	0.233	0.186

**Table A3:** Temperature-dependent thermal conductivities and coefficient of linear expansion of APC2 PEEK (tape).

Temperature T (°C)	Thermal Conductivity k_11_ (W/mK)	Thermal Conductivity k_22_ = k_33_ (W/mK)	Coefficient of Expansion α_11_ (10^−7^ K^−1^)	Coefficient of Expansion α_22_ = α_33_ (10^−5^ K^−1^)
0	3.5	0.42	1.5	2.82
50	4.6	0.52	3.0	2.96
100	5.1	0.6	5.0	3.16
150	5.9	0.7	2.0	3.69
200	5.9	0.7	0	7.3
250	6.1	0.7	0	7.7
300	6.7	0.75	0	8.4
350	6.8	0.68	0	8.8
400	7	0.65	0	8.2

**Table A4:** Elastic and thermal properties of Stainless Steel SS-316 (roller).

Density *ρ*, (kg/m^3^)	Specific Heat C*_p_*, (J/kgK)	Elastic Modulus E, (GPa)	Poisson’s Ratio	Thermal Conductivity k, (W/mK)	Coefficient of Expansion α, (10^−6^ K^−1^)
7760	502	208	0.3	16	7.2
